# Chronic activation profile of circulating CD8+ T cells in Sézary syndrome

**DOI:** 10.18632/oncotarget.23334

**Published:** 2017-12-16

**Authors:** Marina Passos Torrealba, Kelly Cristina Manfrere, Denis R. Miyashiro, Josenilson F. Lima, Luana de M. Oliveira, Nátalli Z. Pereira, Jade Cury-Martins, Juliana Pereira, Alberto J.S. Duarte, Maria N. Sato, José A. Sanches

**Affiliations:** ^1^ Medical Investigation Laboratory (LIM-56), Tropical Medicine Institute of São Paulo, Department of Dermatology, University of São Paulo Medical School, São Paulo, Brazil; ^2^ Cutaneous Lymphoma Clinic, Hospital das Clinicas, Department of Dermatology, University of São Paulo Medical School, São Paulo, Brazil; ^3^ Hematology Department, Hospital das Clinicas, University of São Paulo Medical School, São Paulo, Brazil

**Keywords:** Sézary syndrome, CD8+ T cells, chronic activation markers, sCD38, sCD127

## Abstract

Sézary syndrome (SS) is a leukemic variant of cutaneous T cell lymphoma (CTCL), and the neoplastic CD4+ T cells of SS patients undergo intense clonal proliferation. Although Sézary cells have been studied extensively, studies on adaptive immunity regarding CD8+T cells are scarce. This study aimed to investigate activation marker expression in CD8+ T cells according to the differentiation stages and IL-7/IL7Rα axis responses of patients with SS. Moreover, this study aimed to verify the soluble forms of CD38, sCD127 and IL-7 in serum. Although the SS patients of our cohort had reduced numbers of CD8+ T cells, they exhibited higher percentages of CD8+CD38+ T cells, mainly effector/memory CD8+ T cells, than the control group. In contrast, down-regulated expression of the activation markers CD127/IL-7R and CD26 was found in the CD8+ T cells of SS patients. High serum levels of sCD38 and sCD127 and scarce serum levels of IL-7 were detected, emphasizing the immune activation status of SS patients. Moreover, CD8+ T cells from SS patients exhibited IL-7 unresponsiveness to STAT5 phosphorylation and Bcl-2 expression, and IL-7 priming partially restored IFNγ production. Our findings showed a chronic activation profile of CD8+ T cells, as an attenuated cytotoxic profile and impaired IL-7 responsiveness was observed, suggesting chronic activation status of CD8+ T cells in SS patients.

## INTRODUCTION

Sézary syndrome (SS) is a rare, aggressive leukemic type of cutaneous T cell lymphoma (CTCL) in which malignant T cells are found in skin, blood and lymph nodes [1]. Sézary patients are considered to be in advanced disease stages and have a median survival time of approximately 1 to 5 years [2], with bacterial sepsis being the main cause of death [3]. Impairment of cellular immunity has long been described in SS and contributes to the significant morbidity and mortality associated with infectious complications in SS patients [4]. Defects in innate and adaptive immunity, including altered Toll-like receptor (TLR) activation responses [5], natural killer (NK) cells [6], dendritic cells [7] and T cell-mediated immunity [8], with cytokine signaling pathways described in CTCL.

The constitutive activation of STAT5 and STAT3 has been observed in early and late CTCL stages, respectively [9]. In early stages, IL-2, IL-7 and IL-15 signaling via the JAK1 and JAK3 kinases are believed to be responsible for activating STAT5, while in advanced stages of development, IL-21 autocrine signaling is thought to be important for STAT3 activation [9, 10]. In this context, the deregulated Jak3/STAT3/STAT5 signaling in CTCL cells may contribute to the impairment of co-stimulation with CD8+ T cells.

Co-stimulation via CD26, dipeptidyl peptidase IV, in CD8+ T cells may lead to effector functions induced by cytotoxic effects, preferentially via granzyme B, IFNγ production and Fas ligand expression [11]. However, while the absence of CD26 expression is a useful marker for the diagnosis of CD4+ T cell lymphoma in SS patients [12], their CD8+ T cell profile is unknown.

Some cytokines, including IL-7, is crucial for homeostasis, proliferation and life support of CD8+ T lymphocytes [13]. In CTCL, the number of circulating CD8+ T cells is correlated with a better patient outcome [14], and reduced numbers of tumour-infiltrating CD8+ T cells in biopsied CTCL lesions are related to a lack of response to photopheresis therapy [15] and disease progression [16]. Tumour load can chronically stimulate CD8+ T cells, leading to phenotypic changes and functional impairment [17].

In the current study, we examined the activation/exhaustion status of CD8+ T cells in various differentiation stages, cytokine production, and IL-7 responsiveness. We observed a loss of CD26 and CD127 expression in effector CD8+ T cells that was associated with CD38 expression and dysfunctional IL-7 response in SS, demonstrating the chronic activation status of CD8+ T cells.

## RESULTS

### Chronic activation of circulating CD8+T cells in Sézary patients

Laboratorial characteristics of SS patients are shown in Table 1. All patients were determined to be de novo erythrodermic with intense pruritus and showed at least three laboratorial characteristics of SS, including cell counts>1000/mm^3^; T cell clonality skin and blood; and CD4/CD8 ratios >10%, CD4+CD26– ratios >40% and CD4+CD7– ratios >30%.

**Table 1:** Sézary syndrome patient’s characteristics

Clonality TCR												
Patient	Gender	Age	WBC/mm^3^	Lymphoc./mm^3^	Sezary c.	Blood	Skin	CD4+/mm^3^	CD4+CD26–/mm^3^ (%)	CD4+CD7–/mm^3^ (%)	CD8+/mm^3^	CD4/CD8
1	F	75	16500	10200	+	+	+	9690	7267 (75)	ND	204	47
2	F	55	23280	18830	+	+	+	18077	17715 (98)	17535 (97)	377	48
3	M	56	9420	980	–	+	ND	823	296 (36)	25 (3)	29	28
4	M	70	9510	2200	+	+	–	2068	1861 (90)	ND	66	31
7	M	57	11780	3490	+	+	+	3176	2350 (74)	2604 (82)	174	18
8	M	68	11570	6830	+	+	ND	6251	5376 (86)	5063 (81)	295	22
9	F	69	7370	3260	–	+	+	2543	2060 (81)	585 (23)	242	6
10	M	56	12700	7640	+	+	+	6647	5251 (79)	ND	153	43
11	M	62	16050	8670	+	+	+	8150	4238 (52)	3341 (41)	347	24
13	M	48	44640	21430	+	+	+	20572	4526 (22)	411 (2)	428	48
15	F	60	140870	131010	+	+	+	125767	120736 (96)	120736 (96)	5240	24
16	F	65	10700	3700	+	+	–	3293	2700 (82)	231 (7)	148	22
17	F	70	32060	7370	+	+	+	6706	5968 (89)	4158 (62)	147	45
18	F	76	5280	1160	+	+	+	916	540 (59)	183 (20)	81	11
19	F	58	12520	3090	+	–	–	2472	1508 (61)	49 (2)	247	10
20	F	69	16990	4730	+	+	+	4209	3325 (79)	2147 (51)	94	44
21	M	47	17440	8010	+	–	–	1940	155 (8)	ND	652	3
22	F	57	3100	680	+	+	+	401	56 (14)	80 (20)	68	6
23	M	33	12280	1620	+	+	+	1296	687 (53)	635 (49)	130	10
HD (28)	14/F 14/M	54 (30-85)	ND	ND	ND	ND	ND	ND	ND	ND	ND	ND

F = female, M = male; (+) = presence of Sézary cells ≥ 1000/mm^3^; ND = not done.

Reference value for: WBC 5000–10000/mm^3^; Lymph. 1000–3200/mm^3^; CD4+ 900–3400/mm^3^; CD8+300–1000/mm^3^.

**Figure 1:**
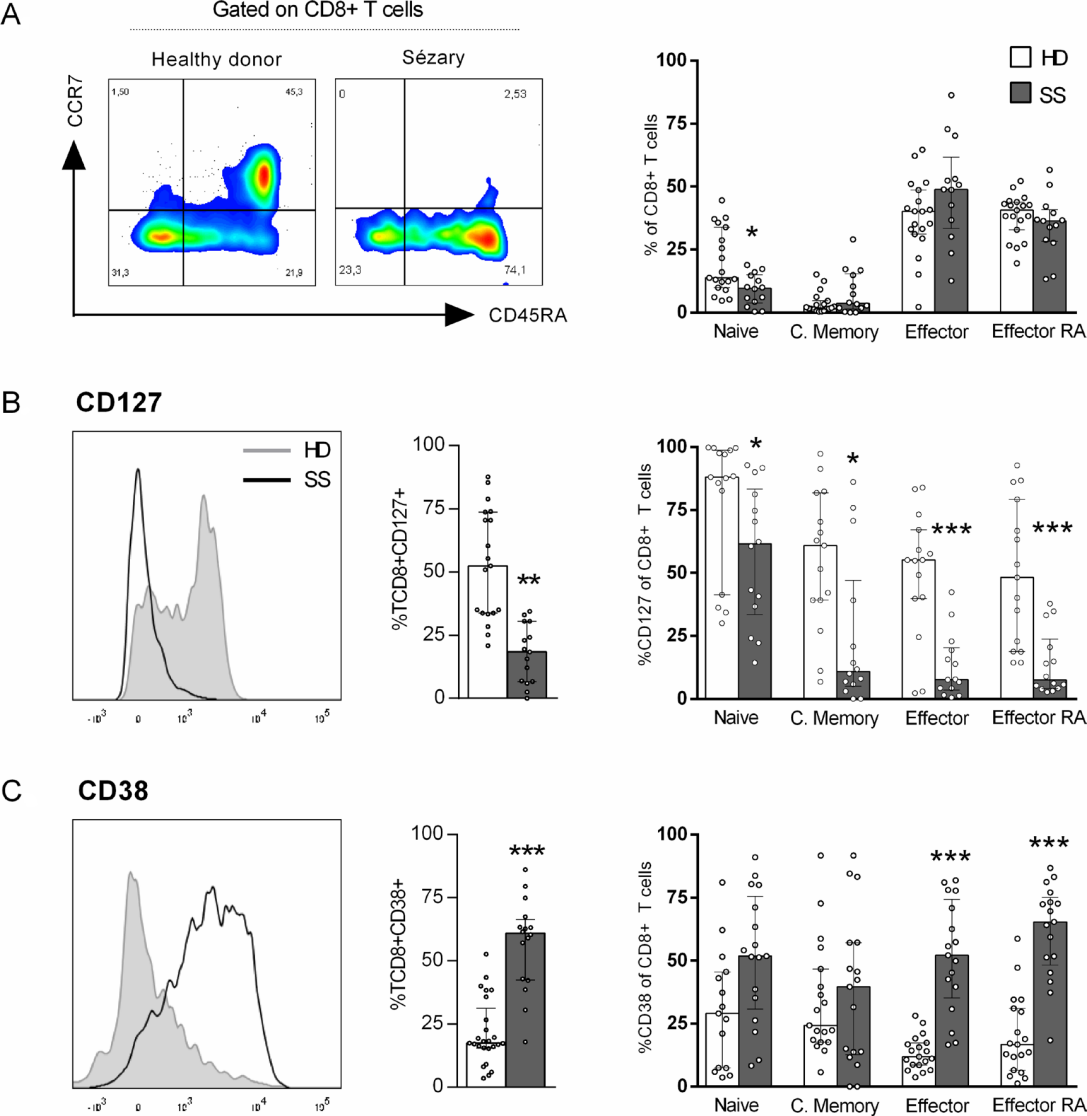
Up-regulation of CD38 in CD8+ T cells from SS patients Peripheral blood CD8+ T cells from SS patients and healthy donors were assessed for memory differentiation subsets and activation markers by flow cytometry. Gating strategy: singlet, lymphocytes, CD3+, CD8+ and memory differentiation. (**A**) CD8+ T cells memory differentiation subsets (*n =* 14 SS and 19 HD), (**B**) Total CD127+CD8+ T cells percentage (*n =* 15 SS and 19 HD) and on CD8+ T cells differentiation subsets (*n =* 14 SS and 15 HD), (**C**) Total CD38+CD8+ T cells percentage (*n =* 16 SS and 25 HD) and on differentiation subset (*n =* 17 SS and 19 HD). The data are shown as median and interquartil. ^*^p ≤ 0.05, ^**^p ≤ 0.01, ^***^p ≤ 0.001.

Reduced numbers of circulating CD8+ T cells have been correlated with the decreased survival probability of CTCL patients [14]. To verify the phenotypic and functional profile of CD8+T cells in SS patients, we first analysed the memory differentiation subsets of peripheral blood CD8+ T cells from Sézary patients and HDs according to their expression of CCR7 and CD45RA Figure 1A shows a reduced percentage of naïve CD8+ T cells in SS patients compared to that in HDs, whereas no differences were observed between the other subsets.

IL-7 signals are vital to T cell development, as they promote the survival of both naïve and memory CD8+ T cells [18]. We verified low percentage of CD127/IL-7α in total and all memory-differentiating CD8+ T cell subsets (Figure 1B). Also, SS patients showed an increased percentage of CD38+, mostly in effector cells (Figure 1C), an activation marker often related to chronic viral infection activation [19].

Moreover, we evaluated exhaustion markers in CD8+ T cells (Supplementary Figure 1), such as PD-1, Tim-3 and CD39, but no differences were verified between the groups.

Taken together, our data provide evidence of a chronic activation profile of circulating CD8+ T cells in Sézary patients.

### Sézary patients exhibit impaired CD26 expression in CD8+ T cells

We assessed CD26 in CD8+ T cells of Sézary patients to verify whether the chronic activation marker CD38 is associated with other activation molecules. The CD26 enzyme is a type II transmembrane glycoprotein that plays a key role in immune regulation as a T cell activation molecule [11].

**Figure 2:**
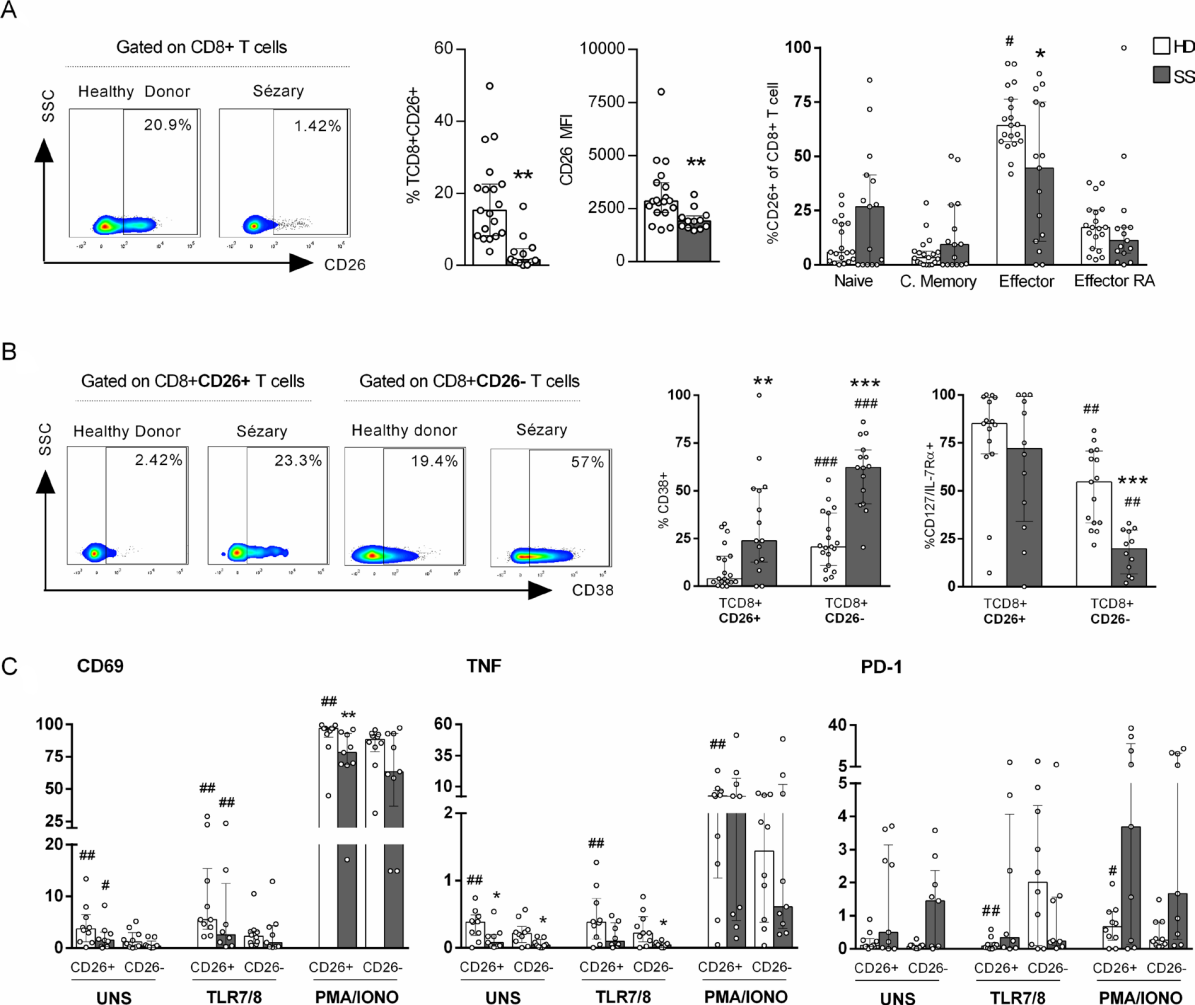
Decreased expression of CD26+ on CD8+ T cells of Sézary patients CD8+ T cells from peripheral blood of SS patients and healthy donors were assessed for CD26+ expression by flow cytometry. PBMC were stimulated by PMA and Ionomycin or TLR 7/8 agonist (CL097). (**A**) Total CD26+ CD8+ T cells percentage, CD26 MFI and memory differentiation of CD8+CD26+ T cells (*n =* 15 SS and 19 HD), (**B**) CD38 and CD127 (*n =* 15 SS and 19 HD) expression on CD8+CD26+ and CD8+CD26- T cells, (**C**) CD69 and PD-1 expression, and TNF production on CD8+ T cells (*n =* 9 SS and 10 HD). The data are shown as median and interquartil. ^*^p ≤ 0.05, ^**^p ≤ 0.01, ^***^p ≤ 0.001 when compared between groups and ^#^ *p* ≤ 0.05, ^##^*p ≤* 0.01 when compared with the same group.

Figure 2A presents reduced numbers of peripheral blood CD8+CD26+ T cells as well as median fluorescence intensity (MFI) levels in SS patients compared to those in HDs. In addition, most of CD8+CD26+ T cells from HDs were effector T cells (Figure 2A) unlike SS group, that CD8+CD26+ T cells were equally distributed between memory differentiation subsets. We verified that the high CD38 expression in SS group were independent of CD26 expression, as were observed in both populations, CD8+CD26+ and CD8+CD26– T cells. However, similarly to both groups, CD38 expression was higher in CD8+CD26– T cells when compared to CD8+CD26+ (Figure 2B). The same phenomenon was observed for CD127/IL-7Rα, as their pronounced decreased expression was observed in CD8+CD26– T cells of SS patients.

Next, we evaluated the expression levels of TNF, CD69 (an early activation marker) and PD-1 (an inhibition receptor) according to CD26 expression in CD8+ T cells upon stimulation. The TLR7/8 agonist was previously shown to partially restore interferon (IFN) responses in CMNs of SS patients [20]. We observed increased CD69 and TNF expression in CD26+ cells compared to that in CD26– cells in the constitutive condition and upon TLR7/8 agonist addition of in both of the groups analysed (Figure 2C). In the unstimulated condition, impaired TNF expression was detected in the SS group regardless of CD26 expression in CD8+ T cells. However, with TLR7/TLR8 stimulation, only CD26– cell numbers were decreased in SS patients, and no differences in IFNγ production or CD107a expression were detected in CD8+ T cells (Supplementary Figure 2).

### Impaired IL-7 signaling in the CD8+ T cells of Sézary patients

**Figure 3:**
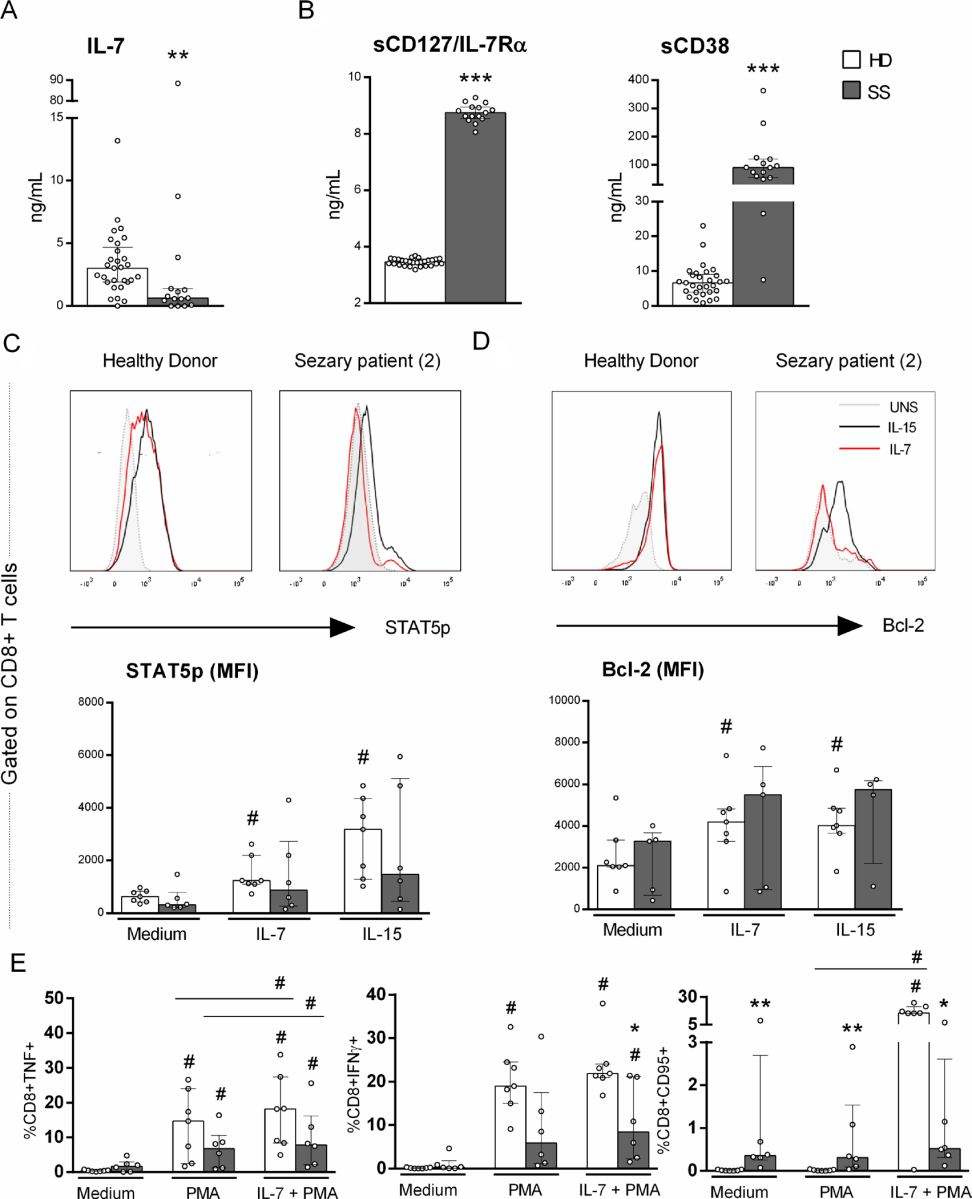
Impaired response to IL-7 of CD8+ T cells from Sézary patients IL-7-signaling on CD8+ T cells from PBMC of SS patients and healthy donors was evaluated for STAT5 phosphorylation, Bcl-2 and CD95/Fas expression and TNF and IFNγ production by flow cytometry. Serum soluble CD127 (IL-7Rα), CD38 and IL-7 were evaluated by ELISA and flow cytometry, respectively (*n =* 15 SS and 28 HD). (**A**) serum IL-7, (**B**) serum sCD127/IL-7Rα and sCD38, (**C**) STAT5p (*n =* 6 SS and 7 HD), (**D**) Bcl-2 expression (*n =* 5 SS and 7 HD) and (**E**) CD95/Fas expression, TNF and IFNγ production induced by PMA and Ionomycin stimulation with previously IL-7 priming (*n =* 6 SS and 7 HD). The data are shown as median and interquartil. ^*^p ≤ 0.05, ^**^p ≤ 0.01, ^***^p ≤ 0.001 when compared between groups and ^#^≤ 0.05 when compared with the same group.

The verified upregulation of CD38 in the CD8+ T cells of Sézary patients together with the altered CD127/IL-7Rα expression led us to evaluate the IL-7 signaling pathway. The serum levels of IL-7 and the soluble form of CD127/IL-7Rα (sCD127) showed opposite trends, as decreased IL-7 levels and increased sCD127 levels were observed in SS patients compared to HDs (Figure 3A and 3B).

Because CD127/IL-7Rα was down-regulated in CD8+ T cells and circulating sCD127 was increased, we analysed IL-7 signaling via STAT5 phosphorylation and Bcl-2 expression. CD8+ T cells from Sézary patients exhibited impaired pSTAT5 and Bcl-2 expression compared to those from HDs (Figure 3C and 3D, respectively), revealing an impaired responsiveness of CD8+ T cells to IL-7 in SS, which could impact their homeostatic function.

Considering that IL-7 can restore lymphocytic functions, such as IFNγ production and proliferation [21], we also evaluated the ability of IL-7 to induce cytotoxic molecules in CD8+ T cells. As expected, CD8+ T cells from SS patients exhibited impaired IFNγ production compared with those of the control group, and IL-7 priming was able to partially restore IFNγ production, albeit at decreased levels compared to that of the HD group.

We demonstrate that unstimulated CD8+ T cells from SS patients showed increased CD95/Fas expression compared to those from HDs (Figure 3E), whereas no IL-7 responsiveness upon the addition of PMA was observed. This finding showed an impaired response to IL-7 in the induction of CD95/Fas in Sézary patients.

## DISCUSSION

Impaired adaptive immune responses are well recognized in SS patients. Despite the reduced numbers of CD8+ T cells in our cohort of SS patients, they exhibited chronic activation profile of CD8+ T cell due to increased CD38 expression, mainly in effector memory cells, as well as attenuated cytotoxic profiles and impaired IL-7 responsiveness. Splitting the memory differentiation stages of peripheral blood CD8+ T cells in SS showed that only the naïve subset was reduced, demonstrating altered homeostatic proliferation during circulation.

CD38 and HLA-DR expression was observed in circulating CD8+ T cells of SS patients and was associated with high serum levels of sCD38, suggesting heightened *immune activation in SS patients.* During chronic viral infections, such as HIV, increased CD38 expression in CD8+ T cells is correlated with disease progression [19] and offers an additional independent predictive value for evaluating plasma viral loads and CD4^+^ T cell counts [22]. Moreover, co-infection with hepatitis C virus and treatment with alpha interferon and ribavirin reduces activation [23]. HIV-1 infection the paradoxical chronic immune activation, leads for co-infection, innate immunity stimulation [24, 25]. In Sézary patients the chronic activation of CD8+ T cells may occur due to the massive proliferation of malignant CD4 + T cells. Majority of the SS patients were naïve of treatment, except two cases (SS19 and SS22). The numbers of CD8+CD38+ T cells in these cases remained elevated (data not shown). This may suggest that similar to patients with chronic viral infections, increased CD38 expression in the CD8+ T cells of SS patients could be correlated with chronic activation.

T cell exhaustion is a state of dysfunction that commonly occurs during chronic infections and cancer due to the persistence of antigens and inflammation [26]. During HIV-1 infection, innate immunity activation via ssRNA TLR7 signaling [27] due to intestinal bacterial translocation and/or co-infection may favor immune activation [28]. After persistent antigen exposure, CD8+ T cells undergo functional loss and exhibit increased expression of co-inhibitory receptors [29, 30]. Their cytotoxicity is modified, and their cytokine production ability, proliferative capacity and effective memory cell generation are also affected [30]. Moreover, HIV+ individuals in late stages of infection have lower numbers of HIV-specific T cell IFNγ producers compared to HIV-specific responses during early HIV infection [31]. This scenario indicates that the accumulation of dysfunctional virus-specific T cells may reduce the functional repertoire of cells accessible for responding to other antigens [32]. Exhausted T cells lose their susceptibility to antigen-independent activation by cytokines, which compromises their ability to detect bacterial co-infections. This could be a factor involved in the SS susceptibility to bacterial sepsis, the main cause of death for these patients [33].

Supporting the chronic activation status of CD8+ T cells in SS patients, decreased CD127 (IL-7R alpha) expression was detected according to the CD8+ T cell maturation stage, and the loss of CD127 expression has been linked to immune activation and reduced numbers of CD4+ T cells during HIV-1 infection [34]. Indeed, sCD38 levels have been shown to be increased during burn shock and significantly decreased post-burn compared to those in healthy volunteers [35]. Until now, sCD38 and sCD127 had not been evaluated in CTCL. The mCD38 expression and the serum levels of total sCD38 are markers of early post-burn lymphocyte activation, and seminal sCD38 is a pivotal regulator of feto maternal tolerance [36]. Whether the increased levels of sCD38 in patients with SS are reflective of immune activation or play a regulatory role requires further investigation.

The IL-7/IL-7R axis plays important roles in the survival and expansion of T cells, and we found increased amounts of sCD127 (IL-7Rα) in serum and reduced IL-7 levels in SS patients. In CTCL, IL-7 and IL-15 have been identified as growth factors, as they can increase the expression of Bcl-2 and the transcription factors STAT2, STAT5, and STAT6 [10]. The decreased IL-7 levels observed in our SS patient cohort may have been due to IL-7 consumption, as most of the patients showed lymphoproliferation of malignant CD4+ T cells. Moreover, the high sCD127 levels should compete with cell-associated CD127, thus reducing IL-7 serum amounts. We verified the impaired CD8+T cell responsiveness to STAT5 and Bcl-2 expression even with *in vitro* IL-7 priming. Increased IL-7 plasma levels have been observed in CTCL patients of stages I-III stage but not in those of stage V [37]. Other diseases with metabolic alterations, such as autoimmune diabetes, have exhibited high serum sCD127 concentrations at the onset of type 1 diabetes [38], showing that metabolic factors may contribute to dysregulation of the IL-7/IL-7 receptor pathway.

Although the loss of CD26 in CD4 T cells is a hallmark of CTCL, our SS patients exhibited a decreased percentage of circulating CD8+CD26+ T cells. CD26, a T cell activation antigen, is a 110kDa cell surface glycoprotein with dipeptidyl peptidase IV activity that inactivates or degrades some bioactive peptides and chemokines [39]. DPPIV/CD26 can regulate cell processes, acting as either a tumour suppressor or tumour activator [40]. The decreased CD8+CD26+ T cell population could reveal the impaired activation of this subset, which comprises mainly memory effector cells. CD26 also has a soluble form, an enzymatically active isoform, which we did not analyse in the SS patients. The decreased CD26 expression exhibited by both unstimulated CD8+TNF+ T cells and CD8+TNF+ T cells activated with TLR7/TLR8 revealed the attenuated status of CD8+ T cells. The loss or alteration of DPPIV expression is linked to the development of several types of cancer, including prostate [41, 42], lung [43], breast [44], hepatocellular carcinoma [45], ovarian [46], and melanoma [47]. However, the increased CD38 expression associated with decreased CD127 and CD26 expression emphasizes the activation profile of CD8+ T cells in SS.

Altogether, our data showed a chronic activation profile of CD8+ T cells with impaired IL-7/IL-7R axis in patients with SS and soluble factors reflecting the commitment of immune effector cells.

## MATERIALS AND METHODS

### Patients

This study included SS patients (*n* = 19, 9 males,10 females) with a median age of 60 years (ranging from 33–76 years) from the Clinic of Cutaneous Lymphomas of the Hospital das Clínicas Department of Dermatology at the University of São Paulo Medical School in Brazil (HCFMUSP). SS diagnoses were established according to the revised clinical, histological, and biological criteria proposed by the International Society for Cutaneous Lymphomas (ISCL) and the cutaneous lymphoma task force of the European Organization of Research and Treatment of Cancer (EORTC). All of the SS patients exhibited “*de novo*” erythrodermia, none of the cases were derived from mycosis fungoides progression. Healthy donors (HDs) (*n =* 28, 14 males, 14 females) with a median age of 54 years (ranging from 30-85 years) were recruited from the Laboratory of Dermatology and Immunodeficiencies (LIM-56). Blood collection was performed before the initiation of treatment (with exception of two cases, that were under treatment), and subjects with dermatological disease or autoimmune disease were excluded from the evaluation. Study exclusion criteria consisted of treatment with immunosuppressant or immune-modifying drugs, pregnancy, and age younger than 18 years. This study was approved by the São Paulo University Institutional Use Committee (CAAE, 07965312.0.0000.0068), and informed consent was obtained from all subjects. All experimental protocols used in this study were performed in accordance to the Ethics Committee of São Paulo University.

### Cell cultures

Mononuclear cells (CMNs) were isolated from heparinized venous blood by Ficoll-Hypaque gradient centrifugation (GE Healthcare Bio-Sciences, Uppsala, Sweden) and diluted in RPMI medium supplemented with 10% AB human serum (Sigma, St Louis, MO, USA). CMNs (2 × 10^6^cells/mL) were incubated with either a TLR7/8 agonist (CL097, 5 µg/mL; Invivogen, San Diego, CA, USA) or phorbol myristate acetate (PMA, 50 ng/mL; Sigma, San Diego, CA, USA), ionomycin (1 µg/mL; Sigma) and CD107a PE-Cy5 (Pe.Cy5/clone H4A3; BD Biosciences, San José, CA, USA) at 37°C and 5% CO_2_. After 6 h of incubation, Brefeldin A (10 µg/mL; Sigma) was added, and the mixtures were incubated for 14 hat 37°C and 5% CO_2_. In some assays, CMNs were primed with rhIL-7 (20 ng/mL; Peprotech, Rocky Hill, NJ, USA) for 24 hr and then stimulated with PMA plus ionomycin. To verify B cell lymphoma 2 (Bcl-2) expression, CMNs were incubated with IL-7 (10 ng/mL) or IL-15 (10 ng/mL; Peprotech) for 48 hours.

### Flow cytometry

To analyse peripheral blood CD8+ T cells, venous blood was collected in EDTA-enriched tubers. Approximately 70 μL of whole blood was stained for 20 minutes and then incubated for 15 minutes with FACS lysing solution (BD Biosciences) to lyse the erythrocytes. Staining was performed using the following antibodies: CD3-BV605 (SK7), CD4-PE-CF594 (RPA-T4), CD8-V500 (RPA-T8), CCR7-AlexaFlour 647 (3D12), CD45RA-APCH7 (HI100), CD26–FITC (M-A261), CD7-Violet 421 (M-T701), CD38-AlexaFluor 700 (HI2), PD1-PE (MIH4), CD127-PeCy7 (HIL-7R-M21) and PDL1-PeCy7 (MIHI). We considered naïve T cells, double-positive cells; CCR7+ single-positive cells were considered central memory; double-negative cells were considered effectors and CD45RA+ single-positive cells were considered terminally differentiated effectors). To analyse T cells after culturing, CMNs were washed and incubated with a LIVE/DEAD Fixable Red Dead Cell Stain kit (Invitrogen, Carlsbad, CA, USA) for 30 min at room temperature. This was followed by fixation with a Cytofix/Cytoperm solution (BD Biosciences) for 20 min and permeabilization with a Perm/Wash solution together with the antibodies for 20 min at 4°C. Staining was performed using the following antibodies: CD3-BV605 (SK7), CD4-V500 (RPA-T4), CD8-AlexaFlur 700 (RPA-T8), CD26–FITC (M-A261), CD69-APC-Cy7 (FN50), IFNγ-V450 (B27), TNF-Pecy7 (Mab11), PD1-PE (MIH4), Bcl-2-FITC (100; Biolegend, San Diego, CA, USA), and CD95-V450 (DX2). For P-STAT5 expression, CMNs were primed with rhIL-7 (1 ng/mL), rhIL-15 (1 ng/mL) or medium for 15 min at 37°C. The cells were labelled according to the manufacturer’s instructions, and analysis was performed by flow cytometry. Staining was performed using the following antibodies: CD3-BV605 (SK7), CD4-APC (RPA-T4), CD8-V500 (RPA-T8) and STAT5-Texas (PY-694). All antibodies were purchased from BD Biosciences. After washing with isotonic solution (Hemoton SPEC; São Paulo, Brazil), 300,000 events were acquired using a flow cytometer (LRSFortessa; BD Biosciences) with FACSDiva software (BD Biosciences). Data were analysed with FlowJo software version 10 (Tree Star, Inc., Ashland, OR, USA).

### Determination of serum IL-7, sCD127and sCD38 levels

Serum samples were collected from venous blood and stored at 80°C until use. Serum IL-7 levels were assessed by flow cytometry using a human IL-7 flex set (BD Biosciences) with a detection limit of 10 pg/mL. Serum sCD127/IL-7R levels were evaluated by an enzyme-linked immunosorbent assay (ELISA) using a human IL7R/CD127 ELISA kit (Life Span, Seattle, WA, USA) with a detection limit of 312 pg/mL. Serum sCD38 levels were evaluated by ELISA using a human CD38 ELISA kit (LifeSpan) with a detection limit of 0.78 ng/mL.

### Statistical analyses

For single comparisons, Mann-Whitney *U*-tests were used to compare variables between groups, and Wilcoxon matched pairs tests were used to compare unstimulated and stimulated sample levels or variables molecule expressions between different populations in the same group. *P*-values ≤ 0.05 were considered significant. Also, Friedman test was used for multiple comparisons between the same group.

### SUPPLEMENTARY MATERIALS FIGURES


