# Supplementary material for: Chronic activation profile of circulating CD8+ T cells in Sézary syndrome
Source: Oncotarget. 2017 Dec 16;9(3):3497–506. doi: 10.18632/oncotarget.23334 (PMC5790478; doi:10.18632/oncotarget.23334)
Supplement: Supplementary file 1 [file oncotarget-09-3497-s001.pdf]

Chronic activation profile of circulating CD8+ T cells in Sézary syndrome

SUPPLEMENTARY MATERIALS

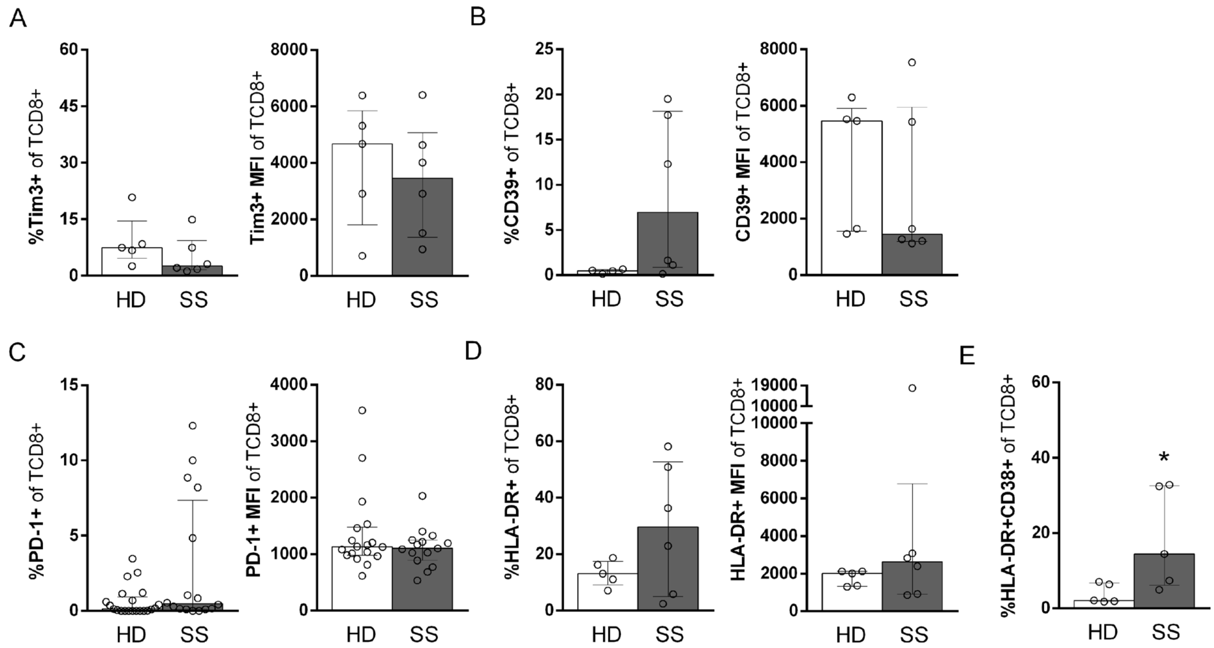

Supplementary Figure 1: Circulating CD8+ T cells from SS patients and healthy donors were assessed for activation and inhibition markers. CD8+ T cells expressing (**A**) Tim3 and MFI (*n* = 6 SS and 5 HD), (**B**) CD39+ and MFI (*n* = 6 SS and 4 HD), (**C**) PD-1+ and MFI (*n* = 17 SS and 21 HD); (**D**) HLA-DR and MFI (*n* = 6 SS and 5 HD) and (**E**) HLA-DR+CD38+ (*n* =6 SS and 5 HD) were assessed by flow cytometry. The data are shown as median and interquartil. $^{*}p \leq 0.05$.

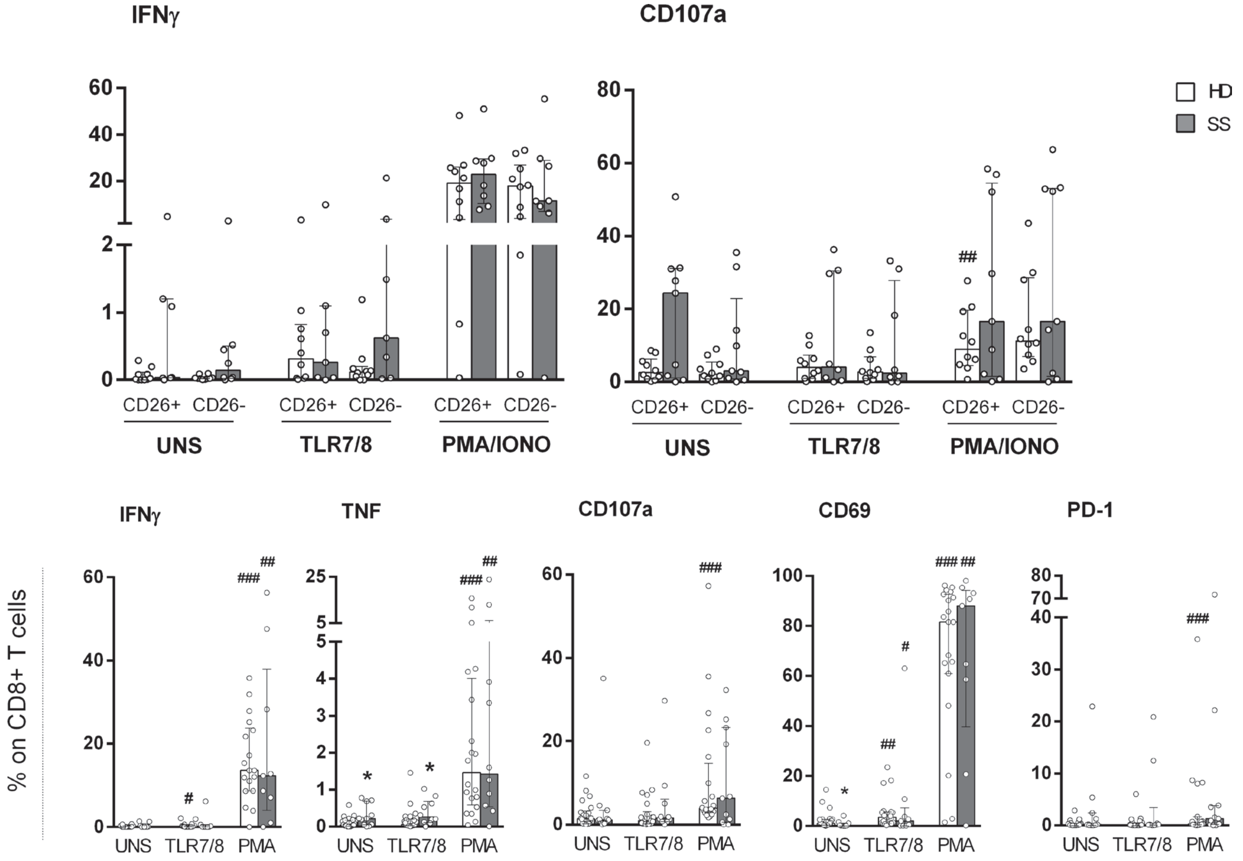

Supplementary Figure 2: Circulating CD8+ T cells from SS patients and healthy donors were assessed for cytokines production and activation. CD8+ T cells expressing (**A**) IFNγ production and CD107a according to CD26+ expression (*n* = 9 SS and 10 HD), (**B**) IFNγ, TNF production and CD69, PD-1 and CD107a were assessed by flow cytometry (*n* = 9 SS and 21 HD). The data are shown as median and interquartil. $^{*}p \leq 0.05$, $^{**}p \leq 0.01$, $^{***}p \leq 0.001$, when compared between groups and $^{\#} \leq 0.05$, $^{\#\#}p \leq 0.01$, $^{\#\#\#}p \leq 0.001$ when compared with unstimulated condition.
